# Accuracy of single breath-hold cine MRI analyzed by guide-point modeling for the assessment of Left Ventricular Function

**DOI:** 10.1186/1532-429X-11-S1-P104

**Published:** 2009-01-28

**Authors:** Christina Heilmaier, Kai Nassenstein, Sonia Nielles-Vallespin, Sven Zuehlsdorff, Peter Hunold, Joerg Barkhausen

**Affiliations:** 1 University Hospital Essen, Essen, Germany; 2 University Hospital, Essen, Germany; 3 Siemens AG Healthcare Sector, MED MR PLM AW Cardiology, Erlangen, Germany; 4 Siemens AG Healthcare Sector, Cardiovascular MR Research and Development, Chicago, IL USA

**Keywords:** Steady State Free Precession, Short Axis View, Volumetric Measurement, Statistical Spread, Steady State Free Precession Sequence

## Purpose

To prospectively assess the performance of highly accelerated cine MRI in multi-orientations combined with a new guide-point modeling post-processing technique (GPM-approach) for the assessment of left ventricular (LV) function compared to the standard summation of slices method based on a stack of short axis views (SoS-approach).

## Materials and methods

33 consecutive patients with sinus rhythm were examined on a 1.5 T scanner with a standard steady state free precession (SSFP) sequence („trueFISP“, TR: 3.0 ms, TE: 1.5 ms, flip angle FA: 60°, matrix: 192 × 156, temporal resolution: 36 ms; acceleration factor AF = 2) in inspiratory breath-hold. End-diastolic volumes (EDV), end-systolic volumes (ESV) and ejection fractions (EF) were calculated from the standard stack of short axis using commercially available software (syngo ArgusVF, version VA80A; SiemensAG Healthcare Sector, Erlangen, Germany). Additionally, 2 long- and 3 short-axis views were measured using a highly accelerated, single breath-hold temporal parallel acquisition SSFP sequence (TPAT; TR: 4.6 ms, TE: 1.1 ms, matrix: 192 × 133, temporal resolution: 40 ms, AF = 3). This data set was analyzed by means of recently implemented software (syngo Argus 4 DVF, version VA80A; Siemens Healthcare Sector, Erlangen, Germany), which builds up a 4-dimensional (4D) model of the left ventricle and allows visualization of the model superimposed to anatomical images as references (guide-point modeling; GPM). In each patient volumetric measurements were performed twice by two independent readers (25 respectively more than 5000 volumetric measurements performed before study was started) using either the stack of short axis approach analyzed with the SoS method or the highly accelerated multi-orientation protocol combined with the GPM technique. For both sequences and post-processing techniques an intra- and interindividual comparison was performed by applying the Bland-Altman approach.

## Results

Mean acquisition and post-processing time was significantly shorter with the GPM-approach (15 seconds/3 minutes versus 360 seconds/6 minutes) when compared to the SoS-method.

### End-Diastolic Volume

Due to an improved definition of the mitral valve plane using the long axis views volumes calculated by the highly accelerated protocol combined with the GPM approach were higher when compared to the stack of short axis views analyzed with the SoS method (mean difference: reader 1, 15.7 ± 11.0 ml; reader 2, 18.6 ± 10.7 ml on average). Moreover, the statistical spread of the mean difference of both readers' EDV was less when measurements were performed with the GPM approach (mean difference 5.7 ± 4.6 ml) compared to the SoS technique (mean difference 8.6 ± 5.9 ml) (Table 1).

**Table 1:** Mean EDV, ESV and EF values ± SD and ranges as determined by reader 1 and 2 with the SoS and GPM approach, respectively

	Stack of short axis views + SoS	Multi-orientation sequences + GPM
**EF**		
R1	53.3% ± 13.5% (range 15–73%)	52.8% ± 13.1% (range 15–71%)
R2	52.8% ± 12.5% (range 16–72%)	53.2% ± 12.6% (range 16–72%)
**EDV**		
R1	148.1 ml ± 57.2 ml (range 85–352 ml)	163.8 ml ± 64.5 ml (range 93–409 ml)
R2	147.4 ml ± 55.0 ml (range 79–331 ml)	165.5 ml ± 63.0 ml (range 98–392 ml)
**ESV**		
R1	75.4 ml ± 58.0 ml (range 23–299 ml)	84.8 ml ± 66.2 ml (range 28–335 ml)
R2	75.5 ml ± 54.3 ml (range 22–274 ml)	84.1 ml ± 63.0 ml (range 28–321 ml)

R2 = reader 2, EDV = end-diastolic volume, ESV = end-systolic volume, EF = ejection fraction, SoS = summation of slices, GPM = guide-point modeling.

### End-Systolic Volume

As with the EDV mean ESV values were higher with the highly accelerated multi-orientation protocol post-processed with the GPM approach for both readers (reader 1, 10.5 ± 10.2 ml; reader 2, 8.8 ± 9.7 ml) and showed less statistical spread in comparison to the stack of short axis views analyzed with the SoS technique (mean difference 6.3 ± 5.0 ml versus 4.3 ± 3.7 ml).

### Ejection Fraction

**Figure 1:**
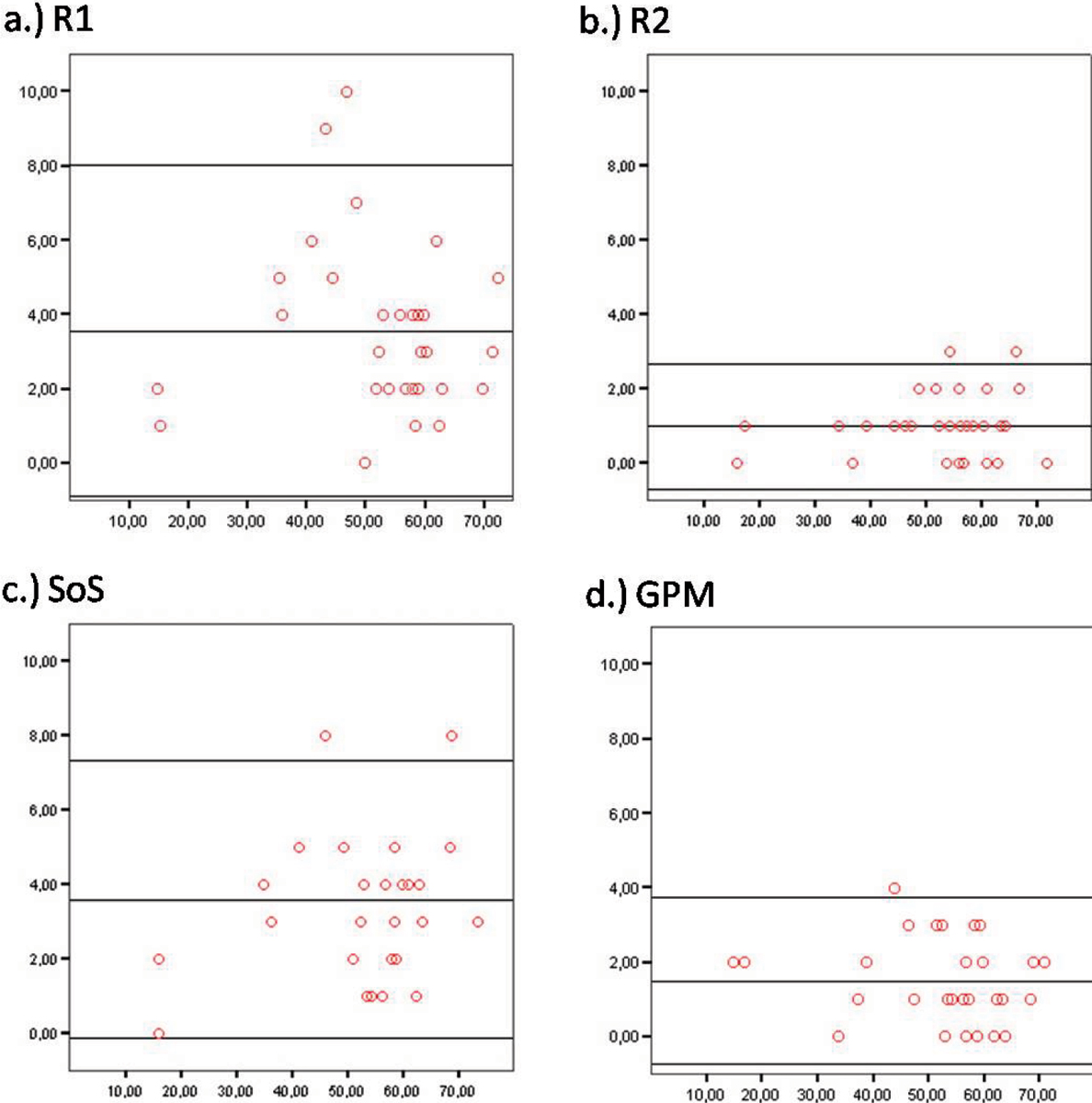
**Bland-Altman-Plot for assessment of ejection fraction**. **(a)** Reader 1 (R1): summation of slices (SoS) versus guide-point modeling (GPM) method. **(b)** Reader 2 (R2): SoS versus GPM approach. **(c)** Interobserver comparison of the SoS method based on a stack of short axis views. **(d**) Multi-orientation highly accelerated sequences analyzed with the GPM approach: R1 versus R2.

EF determined by reader 1 and 2 demonstrated less statistical spread if estimation was performed with the GPM approach (mean difference 1.5 ± 1.1%) compared with the SoS method based on the standard stack of short axis protocols (mean difference 3.6 ± 1.9%). Additionally, the agreement between the EF calculated by the SoS technique and the GPM method was excellent for reader 2 (more than 5000 volumetric measurements performed before study was started; mean difference 1.0 ± 0.9%) and good for reader 1 (25 volumetric measurements done before; mean difference 3.6 ± 2.2%) (Figure 1).

